# Phase Angle Evaluated by a Bioimpedance Analysis Is Closely Related to Diabetic Nephropathy and Peripheral Neuropathy in Patients with Type 2 Diabetes

**DOI:** 10.31662/jmaj.2025-0071

**Published:** 2025-06-27

**Authors:** Hiroyuki Ito, Sayuri Miura, Toshiko Mori, Shun Miura, Chiaki I, Suzuko Matsumoto, Hideyuki Inoue, Shinichi Antoku, Tomoko Yamasaki, Michiko Togane, Moka Sugahara, Chizuko Yukawa

**Affiliations:** 1 Department of Diabetes, Metabolism and Kidney Disease, Edogawa Hospital, Tokyo, Japan; 2 Laboratory Department, Edogawa Hospital, Tokyo, Japan

**Keywords:** bioimpedance analysis, diabetic nephropathy, diabetic neuropathy, extracellular water-to-total body water ratio, phase angle, skeletal muscle mass index, type 2 diabetes

## Abstract

**Introduction:**

The phase angle (PhA), calculated through bioimpedance analysis (BIA), is a measure of skeletal muscle quality and cellular integrity. This study aimed to explore the relationship between PhA and diabetic microangiopathy in patients with type 2 diabetes.

**Methods:**

We conducted a cross-sectional analysis of 556 adult Japanese outpatients with type 2 diabetes who underwent body composition evaluation using BIA. Clinical characteristics, including diabetic microangiopathy (retinopathy, nephropathy, and neuropathy), were assessed for their association with PhA. Furthermore, in 23 patients who underwent a second BIA assessment, we examined the relationship between changes in PhA and glycated hemoglobin (HbA1c) levels longitudinally. Statistical methods employed included Wilcoxon’s signed-rank test, regression analyses, and trend tests.

**Results:**

PhA was significantly lower in patients with diabetic microangiopathy compared to those without it. It was positively correlated with the skeletal muscle mass index (SMI) and negatively correlated with the extracellular water-to-total body water ratio. Multiple regression analysis revealed that sex, age, nephropathy, peripheral neuropathy, hemoglobin, serum albumin, and low-density lipoprotein cholesterol levels were significant explanatory variables for PhA. In the longitudinal analysis, changes in HbA1c levels showed a significant negative correlation with changes in PhA, however, no association was observed between changes in HbA1c and changes in SMI.

**Conclusions:**

PhA is significantly associated with age, malnutrition, and diabetic microvascular complications, and may provide insights into muscle and tissue health in patients with type 2 diabetes. Further research is required to examine causal relationships and explore potential interventions to preserve muscle quality in diabetic patients.

## Introduction

Sarcopenia is commonly observed in patients with type 2 diabetes ^(1)^. Early diagnosis and prevention of sarcopenia are critical for maintaining patients’ quality of life (QOL). The skeletal muscle mass index (SMI), measured using bioimpedance analysis (BIA)―a noninvasive, simple, and cost-effective method for assessing body composition ^(2)^―is one of the diagnostic criteria for sarcopenia ^(3)^.

The phase angle (PhA), also obtained through BIA, serves as an indicator of the integrity of cell membranes and is considered a marker of cellular physiological function in skeletal muscles. When cells are damaged, PhA decreases. Therefore, PhA reflects muscle cell quality, while SMI represents muscle mass ^(4), (5)^. One approach to assessing muscle quality involves measuring muscle strength, but the results can vary depending on the subject’s health condition or motivation during the examination. PhA, by contrast, is an objective parameter that can be calculated using BIA, independent of measurement techniques.

Yamada et al. ^(6)^ reported that muscle mass indicators measured by ultrasonography were lower in groups with sarcopenia and presarcopenia than in other groups. In contrast, muscle quality indicators, measured by a BIA, were lower in groups with sarcopenia and dynapenia (age-related decline in muscle strength prior to the reduction of muscle mass) ^(7)^ than in other groups among 347 elderly individuals from the general population. Barbat-Artigas et al. ^(8)^ found that individuals with high muscle quality―determined by handgrip strength and knee extension strength―had a lower risk of functional impairment, as assessed by the moving, repeat chair stand, and gait speed tests, independent of SMI. On the other hand, individuals with a high SMI but low muscle quality had a high risk of functional impairment among 1,219 women aged ≥75 years. These findings suggest that PhA may be a more sensitive indicator of a subject’s physical function and QOL than SMI.

PhA is generally higher in males than in females ^(9)^. In both sexes, it gradually increases from infancy to adolescence, stabilizes during adulthood, and then decreases with age in older individuals ^(9)^. Low PhA is associated with not only muscle weakness, sarcopenia, and frailty ^(4), (5), (10), (11)^, but also with malnutrition, prolonged hospitalization, and increased mortality in the intensive care unit ^(12), (13), (14)^. It has also been reported that PhA decreases in patients with diabetes compared to non-diabetic individuals ^(15), (16), (17), (18), (19), (20), (21)^.

However, these previous studies did not establish a relationship between PhA and specific clinical factors, including vascular complications. Therefore, we conducted a cross-sectional investigation to explore the associations between PhA and the clinical characteristics of patients with type 2 diabetes, focusing primarily on its relationship with diabetic microangiopathy. However, the effect of glycemic control on PhA could not be discussed in this cross-sectional study. To address this, we examined changes in PhA and glycated hemoglobin (HbA1c) levels in patients who underwent a second body composition evaluation. Examining the relationship between changes in body composition parameters and glycemic control is important, as suggested by this longitudinal study. The results from our investigation will provide insights into the clinical profiles of patients with type 2 diabetes and reduced muscle quality, highlighting those who may require treatment to prevent frailty and sarcopenia.

## Materials and Methods

### Study subjects

This cross-sectional study included 556 adult Japanese outpatients with type 2 diabetes who visited our department between September 2020 and July 2023. The body composition of these patients was evaluated because their attending physician considered it beneficial for treatment. Among them, 23 patients required a follow-up measurement, which was performed at an interval of 418 ± 257 days (median, 441 days).

### Measurements

Body composition was analyzed using BIA with an InBody S10 (InBody Japan Inc., Tokyo, Japan). The following parameters were obtained via BIA: SMI, extracellular water-to-total body water ratio (ECW/TBW), and PhA. Low appendicular skeletal muscle mass was defined as an SMI of <7.0 kg/m^2^ in males and <5.7 kg/m^2^ in females, based on the AWGS (Asian Working Group for Sarcopenia) algorithm for sarcopenia ^(3)^.

PhA was calculated using the formula:

PhA = arctan (*Xc*/*R*)

Reactance (Xc) reflects the opposition to electrical current due to cell membranes and tissue structures, influenced by tissue capacitance. Resistance (R) represents the opposition to alternating current flow through the body, primarily affected by extracellular fluid and tissues. The ratio of Xc to R indicates cell membrane integrity and body water distribution. A higher PhA suggests healthier cell membranes and better hydration, while a lower PhA may indicate impaired cellular integrity or dehydration ^(22)^.

In this study, muscle quality was defined as the functional and structural condition of skeletal muscle tissue, including muscle composition, fat infiltration, membrane integrity, and cellular function ^(7), (23)^. PhA, derived from BIA, reflects the balance between intracellular and extracellular water compartments and cell membrane integrity ^(24)^. Therefore, PhA was used as a surrogate marker of muscle quality.

A current drinker was defined as a person consuming >20 g of ethanol per day. Obesity was defined as a body mass index (BMI) of ≥25.0 kg/m^2^. Hypertension was defined as a systolic blood pressure of ≥140 mmHg and/or a diastolic blood pressure of ≥90 mmHg. Participants using antihypertensive medications were also classified as having hypertension. Hyper-low-density lipoprotein (LDL) cholesterolemia was defined as a serum LDL cholesterol concentration of ≥3.62 mmol/L (140 mg/dL) or current use of statins or ezetimibe. Hypo-high-density lipoprotein (HDL) cholesterolemia was defined as a serum HDL cholesterol concentration of <1.03 mmol/L (40 mg/dL). Hyperuricemia was defined as a serum uric acid level of >327 μmol/L (7.0 mg/dL) or use of urate-lowering agents.

Diabetic retinopathy was diagnosed by the presence of simple, pre-proliferative, or proliferative retinopathy based on the results of a funduscopic examination performed by an expert ophthalmologist. Diabetic nephropathy was defined as a urinary albumin-to-creatinine ratio ≥ 30 mg/g creatinine in a random spot urine test. Diabetic neuropathy was diagnosed by the presence of two or more clinical symptoms (bilateral spontaneous pain, hypoesthesia, or paresthesia of the legs), absence of ankle tendon reflexes, and decreased vibration sensations using a C128 tuning fork ^(25)^. Cerebrovascular disease was diagnosed based on a history of ischemic stroke confirmed by brain computed tomography or magnetic resonance imaging. Coronary heart disease was diagnosed based on a history of myocardial infarction, angina pectoris, or intervention following a coronary angiographic examination. Peripheral artery disease was diagnosed based on obstructive findings on ultrasonographic or angiographic examination of the lower extremities, or an ankle-brachial index <0.9. The estimated glomerular filtration rate (eGFR) was calculated using the formula recommended by the Japanese Society of Nephrology ^(26)^.

### Statistical analyses

All data are presented as the mean ± standard deviation. Wilcoxon’s signed-rank test was used to assess the significance of differences in continuous variables. The χ^2^ test was used for between-group comparisons of categorical variables. The Jonckheere-Terpstra test was used to determine trends in SMI and PhA across groups categorized by age and duration of diabetes. The Cochran-Amitage test was used to determine trends in the percentage of individuals with low appendicular skeletal muscle mass. Wilcoxon’s rank-sum test was used to assess the significance of differences in HbA1c, SMI, ECW/TBW, and PhA during the observation period compared with the respective baseline values. In addition to individual analyses, multiple logistic regression models were constructed to adjust for confounding factors such as age, duration of diabetes, BMI, and HbA1c, to determine the association between PhA and diabetic microangiopathy. Receiver operating characteristic (ROC) analyses were performed to determine the optimal PhA cutoff values for detecting diabetic retinopathy, nephropathy, and peripheral neuropathy. Linear regression analysis using a least-squares model was used to evaluate the associations between clinical parameters and PhA. Factors that showed a significant association with PhA in the single regression analyses were included in the multiple regression analysis. Statistical significance was set at *p* < 0.05 (two-tailed). JMP version 12.2.0 (SAS Institute, Cary, NC, USA) and EZR version 1.68 (Saitama Medical Center, Jichi Medical University, Saitama, Japan), which is a graphical user interface for R (The R Foundation for Statistical Computing, Vienna, Austria), were used to perform all analyses.

## Results

Table 1 shows the clinical characteristics of the study participants. The female group was significantly older than the male group. A history of smoking, current alcohol consumption, and the use of insulin preparations were significantly more prevalent in males, whereas metformin use was more common in females. The prevalence of diabetic retinopathy, nephropathy, and peripheral neuropathy did not differ substantially between males and females; however, coronary heart disease was more frequent in males. Hemoglobin and serum uric acid levels were significantly higher in males than in females, while serum albumin concentrations did not significantly differ between the two groups. In the male subjects, SMI, ECW/TBW, and PhA were 7.82 ± 1.16 kg/m^2^, 0.391 ± 0.013, and 5.33 ± 0.99°, respectively. In the female subjects, SMI, ECW/TBW, and PhA were 6.14 ± 0.94 kg/m^2^, 0.397 ± 0.010, and 4.58 ± 0.70°, respectively. SMI decreased with increasing age in both males and females (Supplementary Figure S1A). The percentage of individuals with low appendicular skeletal muscle mass increased with age (Supplementary Figure S1B).

**Table 1.** Clinical Characteristics of the Subjects.

	N†	%/mean ± SD			
Parameters	556	All subjects	Male	Female	P††
		(n = 556)	(n = 369, 66%)	(n = 187, 34%)	
Age (years)	556	65 ± 14	64 ± 14	69 ± 13	<0.01
Duration of diabetes (years)	514	13 ± 11	12 ± 11	13 ± 11	0.16
Smoking history (%)	550	52	65	28	<0.01
Current drinker (%)	551	32	44	8	<0.01
Body mass index (kg/m^2^)	556	25.8 ± 4.9	26.0 ± 5.0	25.5 ± 4.8	0.25
Concomitant medication (%)					
Sulfonylureas		8	7	9	0.46
Metformin		43	38	52	<0.01
DPP-4 inhibitors		41	39	44	0.25
SGLT2 inhibitors		35	37	30	0.09
GLP-1 receptor agonists		16	17	16	0.70
Insulin		28	31	22	0.02
Obesity	556	52	54	49	0.22
Hypertension	556	78	81	72	0.02
Hyper-LDL cholesterolemia	556	63	59	73	<0.01
Hypo-HDL cholesterolemia	556	21	27	7	<0.01
Hyperuricemia	556	21	27	7	<0.01
Diabetic retinopathy	507	27	27	26	0.83
Diabetic nephropathy	553	53	55	50	0.25
Diabetic peripheral neuropathy	488	43	40	48	0.10
Cerebrovascular disease	556	12	13	10	0.37
Coronary heart disease	556	17	21	10	<0.01
Peripheral artery disease	556	5	5	6	0.52
Systolic blood pressure (mmHg)	531	140 ± 20	139 ± 20	140 ± 20	0.98
Diastolic blood pressure (mmHg)	531	79 ± 13	82 ± 14	75 ± 12	<0.01
Hemoglobin (g/L)	556	140 ± 19	145 ± 19	130 ± 15	<0.01
Serum albumin (g/L)	547	42 ± 4	42 ± 5	42 ± 3	0.18
LDL cholesterol (mmol/L)	537	2.66 ± 0.94	2.64 ± 0.97	2.71 ± 0.89	0.32
HDL cholesterol (mmol/L)	536	1.34 ± 0.37	1.27 ± 0.34	1.47 ± 0.37	<0.01
Serum uric acid (μmol/L)	541	302.4 ± 78.2	318.1 ± 76.4	271.1 ± 72.6	<0.01
eGFR (mL/min/1.73 m^2^)	554	70.4 ± 26.6	69.1 ± 27.4	72.9 ± 24.9	0.11
HbA1c (%)	553	8.5 ± 2.2	8.6 ± 2.3	8.2 ± 1.9	0.07
HbA1c (mmol/mol)	553	69.0 ± 23.8	70.4 ± 24.9	66.1 ± 21.2	0.07
SMI (kg/m^2^)	556	7.25 ± 1.35	7.82 ± 1.16	6.14 ± 0.94	<0.01
ECW/TBW	556	0.393 ± 0.013	0.391 ± 0.013	0.397 ± 0.010	<0.01
PhA (degrees)	556	5.08 ± 0.97	5.33 ± 0.99	4.58 ± 0.70	<0.01

†N: estimated number, ††p: *p*-values for comparisons between male and female groups. DPP: dipeptidyl peptidase; ECW/TBW: extracellular water-to-total body water ratio; eGFR: estimated glomerular filtration rate; GLP: glucagon-like peptide; HDL: high-density lipoprotein; LDL: low-density lipoprotein; PhA: phase angle; RAS: renin-angiotensin system; SGLT: sodium-glucose cotransporter; SMI: skeletal muscle index.

**Figure 1.**
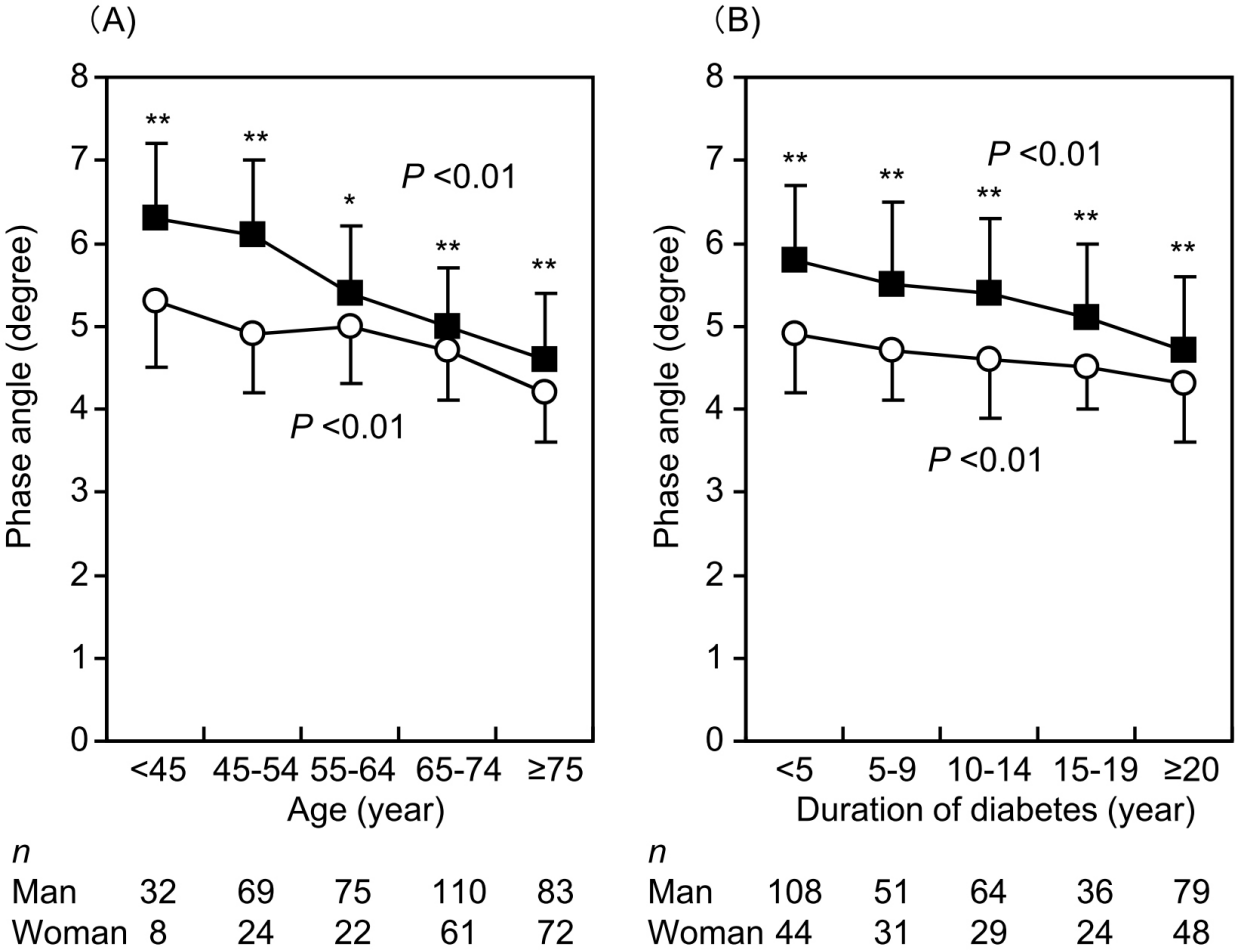
Phase angles according to age (A) and duration of diabetes (B). PhA significantly decreased with increasing age and duration of diabetes in both males and females, (P<0.01, respectively, Jonckheere-Terpstra test). Closed squares and open circles indicate males and females, respectively. **p <0.01 and *p <0.05 vs. female (Wilcoxon’s rank-sum test).

PhA significantly decreased with increasing age (Figure 1A) and duration of diabetes (Figure 1B) in both males and females (p < 0.01, respectively). PhA was higher in males than in females across all age groups. HbA1c levels decreased with increasing age (Supplementary Figure S2).

Table 2 compares the age, duration of diabetes, and parameters obtained by BIA between patients with and without diabetic microangiopathy. Although differences were not always significant, both males and females with microangiopathy were older and had a longer duration of diabetes than those without microangiopathy. SMI was significantly lower in male patients with diabetic peripheral neuropathy than in those without neuropathy. However, no statistically significant difference was found between patients with and without diabetic retinopathy or nephropathy. Furthermore, in female subjects, SMI did not differ significantly between patients with and without diabetic microangiopathy. In both sexes, PhA was significantly lower in patients with any form of microangiopathy than in those without, whereas ECW/TBW was significantly higher in those with any microangiopathy.

**Table 2.** Patient Age, Duration of Diabetes, and Body Composition Parameters of Patients with and without Diabetic Microangiopathy.

Parameters	Retinopathy			Nephropathy			Neuropathy		
	(+)	(−)	p	(+)	(−)	p	(+)	(−)	p
Male									
Age (years)	67 ± 12	62 ± 14	<0.01	64 ± 13	62 ± 14	0.20	68 ± 11	61 ± 15	<0.01
Duration (years)	18 ± 11	11 ± 11	<0.01	13 ± 12	11 ± 11	0.14	17 ± 13	9 ± 9	<0.01
SMI (kg/m^2^)	7.62 ± 1.24	7.84 ± 0.99	0.09	7.90 ± 1.29	7.72 ± 0.96	0.42	7.53 ± 0.93	7.99 ± 1.13	<0.01
ECW/TBW	0.396 ± 0.013	0.388 ± 0.012	<0.01	0.393 ± 0.014	0.387 ± 0.012	<0.01	0.395 ± 0.012	0.387 ± 0.012	<0.01
PhA (degrees)	4.90 ± 0.86	5.53 ± 0.95	<0.01	5.18 ± 1.00	5.53 ± 0.95	<0.01	4.96 ± 0.87	5.65 ± 0.89	<0.01
Female									
Age (years)	72 ± 12	67 ± 14	0.01	70 ± 14	68 ± 13	0.18	71 ± 12	67 ± 14	0.03
Duration (years)	16 ± 12	12 ± 10	0.06	15 ± 11	12 ± 11	0.18	16 ± 11	11 ± 10	<0.01
SMI (kg/m^2^)	5.93 ± 1.00	6.21 ± 0.93	0.08	6.16 ± 1.00	6.13 ± 0.89	0.80	6.08 ± 0.96	6.10 ± 0.93	0.75
ECW/TBW	0.401 ± 0.009	0.396 ± 0.010	<0.01	0.399 ± 0.009	0.395 ± 0.010	0.01	0.400 ± 0.009	0.395 ± 0.009	<0.01
PhA (degrees)	4.36 ± 0.73	4.66 ± 0.68	0.01	4.48 ± 0.75	4.68 ± 0.63	0.04	4.43 ± 0.68	4.68 ± 0.70	0.02

ECW/TBW: extracellular water-to-total body water ratio; PhA: phase angle; SMI: skeletal muscle index.

Table 3 presents the odds ratios for PhA in relation to diabetic microangiopathy in male and female groups. In both sexes, a lower PhA was significantly associated with the presence of diabetic retinopathy, nephropathy, and peripheral neuropathy in single logistic regression analyses. After adjusting for age, duration of diabetes, BMI, and HbA1c, PhA remained significantly associated with diabetic microangiopathy. The ROC curve analysis identified clinically relevant PhA cutoff values for detecting diabetic retinopathy, nephropathy, and peripheral neuropathy in male subjects as 5.3°, 5.9°, and 5.6°, respectively (Supplementary Figure S3). In female subjects, the corresponding cutoff values were 4.2°, 4.4°, and 4.6°, respectively.

**Table 3.** Odds Ratios of Phase Angle for Diabetic Microangiopathy.

Microangiopathy	OR [95% CI]	*p*	OR [95% CI]†	*p*
Male				
Retinopathy	0.48 [0.36-0.63]	<0.01	0.50 [0.35-0.73]	<0.01
Nephropathy	0.69 [0.55-0.86]	<0.01	0.64 [0.46-0.86]	<0.01
Neuropathy	0.41 [0.30-0.54]	<0.01	0.51 [0.34-0.73]	<0.01
Female				
Retinopathy	0.52 [0.31-0.86]	0.01	0.64 [0.34-1.18]	0.15
Nephropathy	0.65 [0.42-0.99]	0.04	0.59 [0.34-1.01]	0.06
Neuropathy	0.59 [0.37-0.93]	0.02	0.77 [0.43-1.38]	0.38

†OR adjusted for age, duration of diabetes, body mass index, and HbA1c CI: confidence interval; OR: odds ratio.

**Figure 2.**
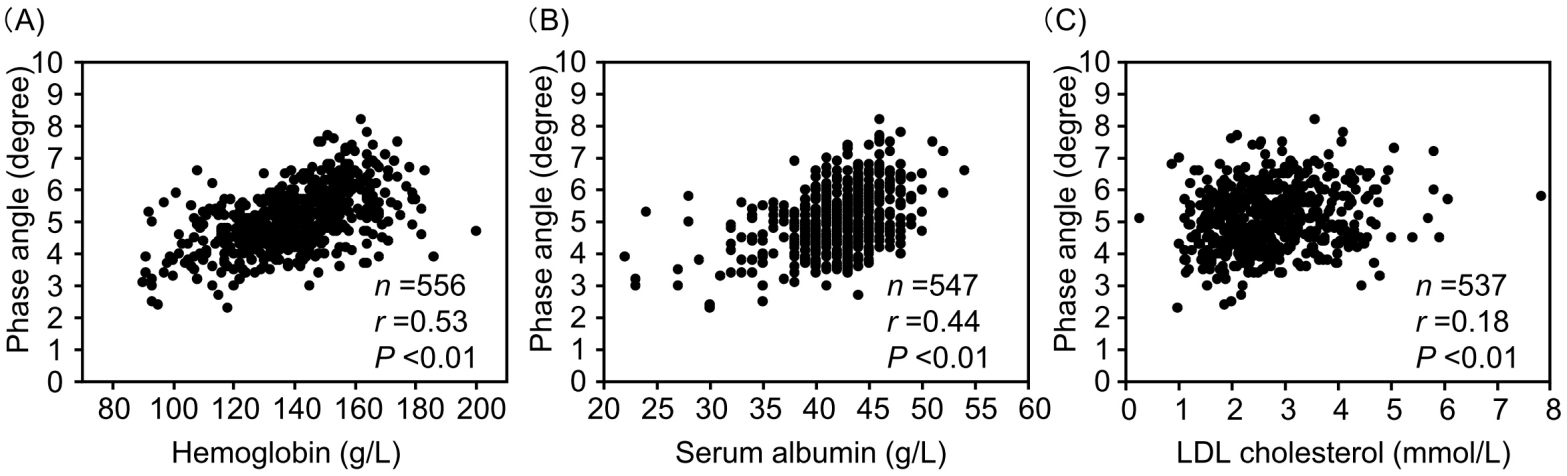
Relationships between hemoglobin (A), serum albumin (B), and LDL cholesterol (C), and the phase angle.

**Figure 3.**
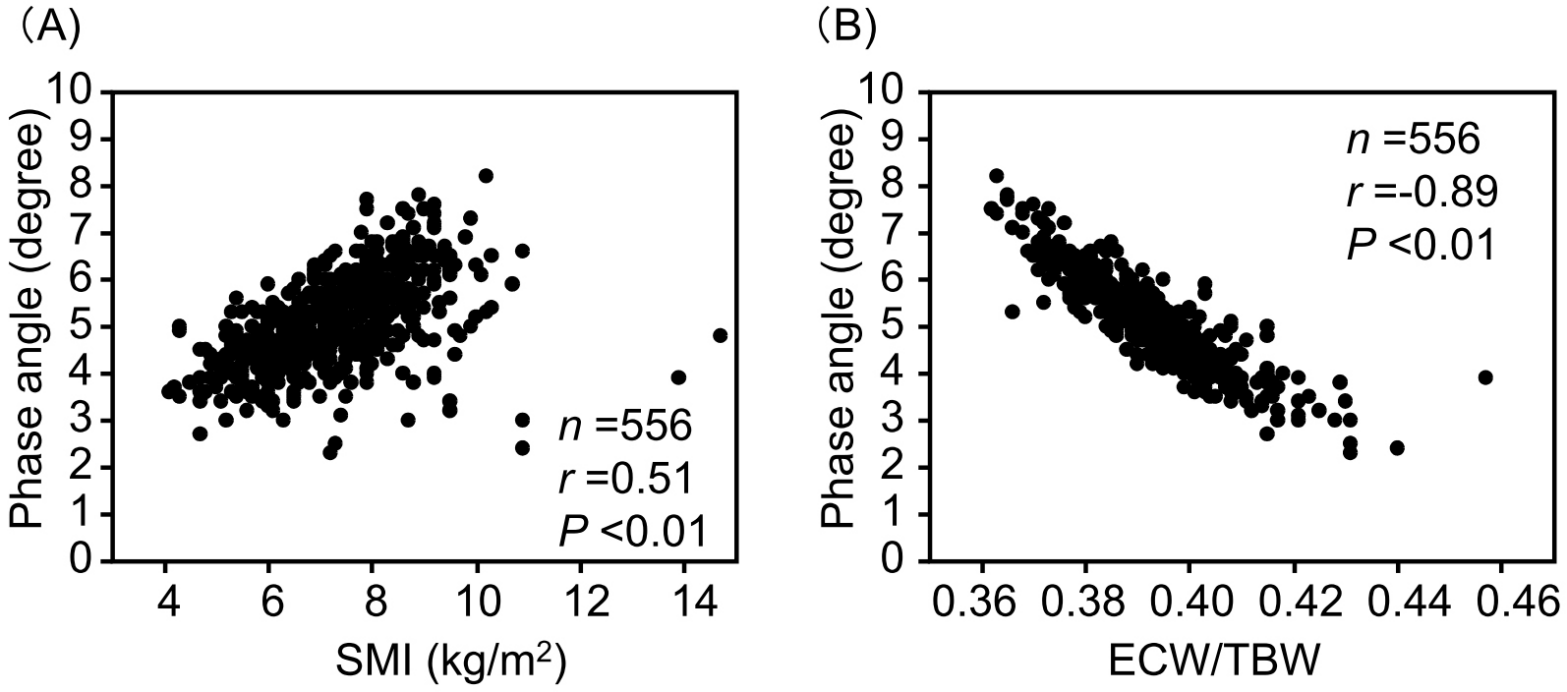
Relationships between the SMI (A) and ECW/TBW (B) and the phase angle. ECW/TBW, extracellular water-to-total body water ratio; SMI, skeletal muscle index.

PhA showed significant positive correlations with hemoglobin, serum albumin, and LDL cholesterol levels (Figure 2). Additionally, PhA demonstrated a significant positive correlation with SMI and a negative correlation with ECW/TBW (Figure 3). Since sodium-glucose cotransporter 2 (SGLT2) inhibitors may affect body composition ^(27)^, the relationship between PhA and SMI or ECW was analyzed in 193 SGLT2 inhibitor users and 363 non-users (Supplementary Figure S4). PhA showed a significantly positive correlation with SMI in both SGLT2 inhibitor users and non-users. PhA also showed a significantly positive correlation with ECW/TBW in both SGLT2 inhibitor users and non-users.

Table 4 shows the association between PhA and clinical characteristics. Due to multicollinearity between PhA and ECW/TBW from the standardized regression coefficients in the single regression, ECW/TBW was excluded from the independent variables in multiple regression analyses. In a multiple regression analysis (model 1), age, BMI, diabetic nephropathy, peripheral neuropathy, hemoglobin, serum albumin, eGFR, and SMI were significant explanatory variables for PhA. In model 2, after excluding SMI from the independent variables (as it was clearly confounded with PhA), sex, age, diabetic nephropathy, peripheral neuropathy, hemoglobin, serum albumin, LDL cholesterol levels, and eGFR were significant explanatory variables for PhA.

**Table 4.** Associations between Phase Angle and the Clinical Characteristics.

Parameters	Single regression		Multiple regression			
			Model 1		Model 2	
	β	p	β	p	β	p
Male	0.756	<0.01	−0.019	0.88	0.442	<0.01
Age (/years)	−0.040	<0.01	−0.023	<0.01	−0.030	<0.01
Duration of diabetes (/years)	−0.031	<0.01	0.003	0.35	0.002	0.61
Smoking history	0.224	<0.01	0.041	0.56	0.034	0.64
Current drinker	0.436	<0.01	0.032	0.67	0.018	0.81
Body mass index (/kg/m^2^)	0.061	<0.01	−0.029	0.03	0.012	0.31
Concomitant medication						
Sulfonylureas	−0.384	0.01	−0.080	0.46	−0.077	0.50
Metformin	0.103	0.22				
DPP-4 inhibitors	−0.401	<0.01	0.080	0.24	0.049	0.49
SGLT2 inhibitors	0.344	<0.01	0.079	0.25	0.098	0.16
GLP-1 receptor agonists	0.208	0.06				
Insulin	−0.074	0.42				
Obesity	0.590	<0.01	0.125	0.19	0.119	0.22
Hypertension	−0.350	<0.01	−0.102	0.27	−0.121	0.21
Hyper-LDL cholesterolemia	−0.076	0.37				
Hypo-HDL cholesterolemia	0.237	0.02	0.037	0.71	0.018	0.86
Hyperuricemia	0.037	0.72				
Diabetic retinopathy	−0.510	<0.01	−0.096	0.21	−0.113	0.16
Diabetic nephropathy	−0.261	<0.01	−0.167	<0.01	−0.178	<0.01
Diabetic peripheral neuropathy	−0.598	<0.01	−0.235	<0.01	−0.236	<0.01
Cerebrovascular disease	−0.532	<0.01	−0.039	0.71	−0.149	0.17
Coronary heart disease	−0.255	0.02	−0.063	0.47	−0.055	0.17
Peripheral artery disease	−0.497	<0.01	−0.001	1.00	−0.036	0.82
Systolic blood pressure (/mmHg)	−0.005	0.02	−0.001	0.62	0.000	0.93
Diastolic blood pressure (/mmHg)	0.020	<0.01	0.000	0.92	−0.001	0.74
Hemoglobin (/g/L)	0.028	<0.01	0.006	<0.01	0.006	0.03
Serum albumin (/g/L)	0.101	<0.01	0.059	<0.01	0.055	<0.01
LDL cholesterol (/mmol/L)	0.190	<0.01	0.067	0.07	0.076	0.049
HDL cholesterol (/mmol/L)	−0.530	<0.01	−0.067	0.52	−0.062	0.57
Serum uric acid (/μmol/L)	0.002	<0.01	−0.000	0.56	0.000	0.44
eGFR (/mL/min/1.73 m^2^)	0.009	<0.01	−0.005	<0.01	−0.006	<0.01
HbA1c (/%)	0.038	0.04	0.032	0.06	0.019	0.28
SMI (/kg/m^2^)	0.368	<0.01	0.295	<0.01		
ECW/TBW	−67.979	<0.01				

Abbreviations: DPP, dipeptidyl peptidase; ECW/TBW, extracellular water-to-total body water ratio; eGFR, estimated glomerular filtration rate; GLP, glucagon-like peptide; HDL, high-density lipoprotein; LDL, low-density lipoprotein; RAS, renin-angiotensin system; SGLT, sodium-glucose cotransporter; SMI, skeletal muscle index

Supplementary Figure S5 shows the relationships between changes in HbA1c values and parameters obtained by BIA in the 23 patients who underwent a second body composition evaluation during the diabetes treatment period. At the first evaluation, HbA1c, SMI, ECW/TBW, and PhA were 9.3 ± 2.5%, 7.06 ± 1.33 kg/m^2^, 0.392 ± 0.011, and 5.01 ± 0.80°, respectively. At the second evaluation, HbA1c levels significantly improved to 7.9 ± 1.4% (p = 0.02). The SMI (7.07 ± 1.30 kg/m^2^, p = 0.98), ECW/TBW (0.394 ± 0.011, p = 0.09), and PhA (4.93 ± 1.01°, p = 0.06) values were not significantly different from the corresponding values at the first evaluation. Although the change in HbA1c was significantly positively correlated with the change in ECW/TBW (Supplementary Figure S5B) and negatively correlated with the change in PhA (Supplementary Figure S5C), it was unrelated to the change in SMI (Supplementary Figure S5A).

## Discussion

The present study is the first to show a significant association between PhAs obtained using BIA and diabetic nephropathy and peripheral neuropathy in patients with type 2 diabetes. This novel finding clarifies the clinical characteristics of patients with decreased PhA levels, particularly its association with diabetic microangiopathy. Previous studies have reported a decrease in PhA in patients with type 2 diabetes ^(11), (12), (13), (14), (15), (16), (17)^. In the present study, however, no significant difference was found in SMI between patients with and without retinopathy or nephropathy. As PhA is considered superior to SMI in terms of sensitivity to the physical function ^(7), (8)^, these findings are important for the early detection of sarcopenia in patients.

The relationship between PhA and diabetic nephropathy may be explained by the strong negative correlation observed between PhA and ECW/TBW. This is consistent with previous studies in patients with diabetes ^(21), (28), (29), (30), (31), (32)^. ECW/TBW increases even in healthy individuals due to decreased intracellular water with aging ^(33)^. However, in the present study, the association between PhA and diabetic nephropathy likely reflects a decline in muscle quality due to excess extracellular water, as ECW/TBW increases in patients with chronic kidney disease, reflecting water retention. This alteration in water balance is particularly emphasized in patients with diabetes, even in stages without peripheral edema ^(20)^.

Since ECW/TBW and PhA are derived from the same BIA measurements, it is not surprising that they are inversely correlated, as seen in the present study. An elevated ECW/TBW ratio reflects extracellular fluid expansion, inflammation, and endothelial dysfunction, all of which can contribute to microvascular damage in patients with diabetes ^(2), (22), (24), (34)^. In contrast, PhA, which reflects cell membrane integrity and body cell mass, is independently associated with nutritional and functional status and has predictive value for clinical outcomes ^(2), (22), (24), (34)^. The relationship between ECW/TBW and diabetic microangiopathy may partially overlap with that of PhA. However, these two parameters represent distinct physiological aspects: ECW/TBW primarily reflects fluid distribution abnormalities, while PhA directly indicates cellular health and membrane integrity. Both indices are clinically relevant, providing complementary insights into the pathophysiology of diabetic microangiopathy. Further studies are required to clarify the precise mechanistic pathways underlying these associations.

Schimpfle et al. ^(35)^ recently proposed that PhA is a reliable marker for detecting diabetic neuropathy, as PhA significantly correlated with the conduction velocity and amplitude of the peroneal and tibial nerves in 104 healthy subjects and 205 patients with type 2 diabetes, including 63 individuals with neuropathy. Zhang et al. ^(36)^ also reported that PhA was inversely correlated with the risk of diabetic neuropathy, based on the vibration perception threshold in 697 patients with type 2 diabetes. These findings are easily understood, considering the frequent occurrence of muscle atrophy and sarcopenia in patients with diabetic neuropathy ^(37), (38)^.

In the cross-sectional study, PhA was independent of HbA1c levels. Previous studies have reported a negative correlate between PhA with HbA1c and/or fasting plasma glucose levels in patients with diabetes ^(15), (16), (19), (28), (31), (39)^. This negative correlation is thought to be due to the alteration in osmolarity of extracellular components induced by hyperglycemia and the reduction in metabolically active cell mass ^(40)^. This result suggests that reduced PhA reflects muscle cell death or degeneration caused by the movement of water from intracellular to extracellular spaces due to hyperglycemia. However, some reports indicate that PhA and HbA1c are unrelated ^(29), (30), (32), (41)^. Notably, HbA1c levels by age group were not described in those studies. In our study, HbA1c levels were higher in younger subjects who generally had higher PhA levels than older subjects (Supplementary Figure S1). This likely explains why no relationship between PhA and glycemic control status was observed in our study. Conversely, the change in PhA was significantly negatively correlated with changes in HbA1c levels in patients whose body composition was re-evaluated. However, changes in HbA1c did not significantly correlate with changes in SMI. This suggests that hyperglycemia management may improve or maintain muscle quality independently of muscle mass, even though PhA, which represents muscle quality, typically shows a positive correlation with SMI, which indicates muscle mass. Therefore, we recommend evaluating both PhA and SMI simultaneously when assessing body composition using BIA. Jun et al. ^(18)^ reported that PhA remained independent of short-term fluctuations in blood glucose levels during a meal tolerance test. Therefore, it is advisable to re-evaluate PhA after a certain period; however, the interval required to assess changes in muscle quality remains unknown.

The findings are consistent with previous studies ^(28), (29), (30), (32)^, which showed a significant association between hemoglobin and serum albumin levels and PhA, irrespective of age or sex. The positive correlation observed between PhA and serum LDL cholesterol concentration in this study may also be influenced by the nutritional status of the subjects.

Regarding the relationship between PhA and antidiabetic medication, Mat et al. ^(17)^ reported that male patients using oral antidiabetic agents had lower PhA values in a cohort of 1,085 patients with diabetes, with an average age of 68.11 years. Similarly, Hori et al. ^(30)^ reported that insulin users had lower PhA values in 371 diabetic patients, with an average age of 68 years. In contrast, Dittmar et al. ^(39)^ found no association between PhA and the prescription status of antidiabetic agents in 182 patients with type 2 diabetes. In the current study, single linear regression analyses revealed that PhA was negatively associated with the use of dipeptidyl peptidase (DPP)-4 inhibitors and positively associated with SGLT2 inhibitors. The selection of antidiabetic agents is strongly influenced by factors such as the patient’s age and diabetes duration, which impact BMI, endogenous insulin secretion, and renal function in clinical practice ^(42)^. DPP-4 inhibitors are commonly prescribed to elderly patients, while SGLT2 inhibitors are more often used in non-elderly patients in Japan ^(43), (44), (45)^. In our study, DPP-4 inhibitor users were significantly older than non-users (70 ± 13 years vs. 62 ± 14 years, *p* < 0.01), whereas SGLT2 inhibitor users were significantly younger than non-users (62 ± 13 years vs. 67 ± 14 years, *p* < 0.01). Therefore, when examining the relationship between PhA and diabetes treatment methods, it is important to consider the clinical background of the subjects, including age and diabetes duration.

This study has several limitations. First, it employed a cross-sectional design, which limits our ability to establish causal relationships between PhA and diabetic microangiopathy. Additionally, the study population may not be representative of the entire type 2 diabetes population due to potential selection bias, as the subjects were patients selected based on the attending physicians’ judgment for body composition evaluation. Second, the associations between changes in SMI, ECW/TBW, and PhA and changes in HbA1c during the observation period were derived from a relatively small sample. Thus, these results should be interpreted cautiously due to the limited statistical power of the study. However, despite these limitations, the findings offer valuable insights into the impact of blood glucose management on patient prognosis. Glycemic control appears to influence PhA, but definitive conclusions cannot be drawn at this time. Further research with a larger sample size is needed to validate our findings. Nevertheless, we believe that the results of this study contribute to a deeper understanding of the relationship between muscle quality, QOL, and clinical factors such as glycemic control, vascular complications, and nutritional status in patients with type 2 diabetes.

In conclusion, PhA is significantly associated with age, malnutrition, and diabetic microvascular complications, and may reflect aspects of muscle and tissue health in patients with type 2 diabetes. Further research is necessary to explore causal relationships and potential interventions to preserve muscle quality in diabetic patients.

## Article Information

### Conflicts of Interest

Hiroyuki Ito has received lecture fees from Novo Nordisk Pharma Ltd., Eli Lilly Japan KK, Sanofi KK, Astellas Pharma, Kowa Company, Ltd., Taisho Pharmaceutical Co., Ltd., Sumitomo Pharma Co., Ltd., Boehringer Ingelheim, Daiichi Sankyo Company, Novartis Pharma KK, Takeda Pharmaceutical Company Ltd., MSD KK, Terumo Corporation, Mochida Pharmaceuticals, Teijin Pharma, Kissei Pharmaceuticals, Mitsubishi Tanabe Pharma Corporation, Sanwa Kagaku Kenkyusho, AstraZeneca KK, Kyowa Kirin Co. Ltd., Otsuka Pharmaceutical Co., Ltd., Bayer Yakuhin, Ltd., EA Pharma Co., Ltd., Ono Pharmaceutical Co., Ltd., Viatris Inc., and has received consulting fee from Becton, Dickinson, and Company. Toshiko Mori has received lecture fees from Novartis Pharma KK. Suzuko Matsumoto has received lecture fees from Eli Lilly Japan KK, Novo Nordisk Pharma Ltd., Astellas Pharma, Kyowa Kirin Co., Ltd., and AstraZeneca KK. Hideyuki Inoue has received lecture fees from Novartis Pharma KK, AstraZeneca KK and Mochida Pharmaceuticals. Shinichi Antoku has received lecture fees from Kyowa Kirin Co. Ltd., Sanofi KK, Taisho Pharmaceutical Co., Ltd., Daiichi Sankyo Company, Novo Nordisk Pharma Ltd., Novartis Pharma KK, AstraZeneca KK and Otsuka Pharmaceutical Co., Ltd. Sayuri Miura, Shun Miura, Chiaki I, Tomoko Yamasaki, and Michiko Togane, Moka Sugahara, and Chizuko Yukawa have no conflict of interest.

### Acknowledgement

The authors thank Tomoko Koyanagi in the secretarial section of Edogawa Hospital for her valuable help with data collection.

### Author Contributions

Conception, design, analysis, interpretation, writing of the first draft, editing, and final approval: Hiroyuki Ito. Conception, interpretation, editing, and final approval: Sayuri Miura, Toshiko Mori, and Chizuko Yukawa. Data collection and final approval: Shun Miura, Chiaki I, Suzuko Matsumoto, Tomoko Yamasaki, Michiko Togane, and Moka Igarashi. All the authors have agreed to the final version of the manuscript.

### Approval by Institutional Review Board (IRB)

The study was conducted in accordance with the principles outlined in the Declaration of Helsinki. The Ethics Committee of Edogawa Hospital approved the study protocol and waived the need for written informed consent, as the data were analyzed anonymously for this retrospective analysis, using information stored at the hospital (approved number: 2023-40, approval date: November 6, 2023). The trial was registered with the UMIN-CTR (identifier UMIN000053298)

### Data Sharing

The datasets generated during the current study are available from the corresponding author on reasonable request.

## Supplement

Supplementary Materials
