# Supplementary material for: Phase Angle Evaluated by a Bioimpedance Analysis Is Closely Related to Diabetic Nephropathy and Peripheral Neuropathy in Patients with Type 2 Diabetes
Source: JMA J. 2025 Jun 27;8(3):925–35. doi: 10.31662/jmaj.2025-0071 (PMC12328276; doi:10.31662/jmaj.2025-0071)
Supplement: Supplementary Materials [file 2433-3298-8-3-0925-s001.pdf]

Supplementary material

Supplementary Fig. S1: SMI and rate of individuals with a low appendicular skeletal muscle mass in the study subjects according to age groups.

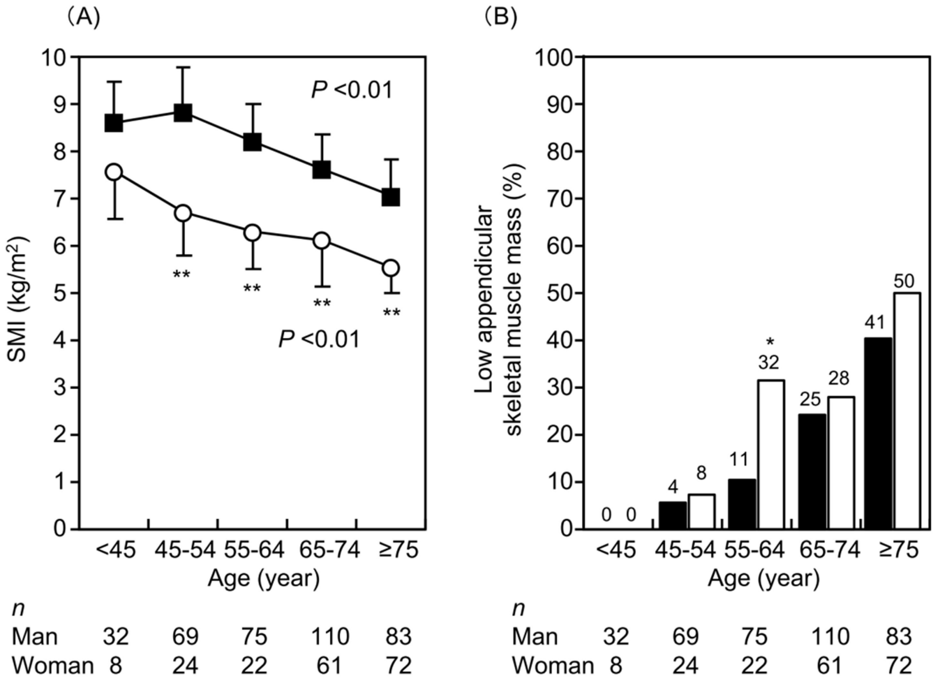

(A) SMI in the study subjects according to age groups. SMI significantly decreased with increasing age in both males and females, (P<0.01, respectively, Jonckheere-Terpstra test). Closed squares and open circles indicate males and females, respectively. ** P <0.01 vs. males (Wilcoxon's rank-sum test).

(B) Rate of individuals with a low appendicular skeletal muscle mass. They increased with age increasing (P<0.01 in males and P=0.08 in females, respectively, Cochran-Amitage test). Closed and open bars indicate males and females, respectively. * P <0.05 vs. males (χ^2 test).

Supplementary Fig. S2: HbA1c values in the study subjects according to age groups.

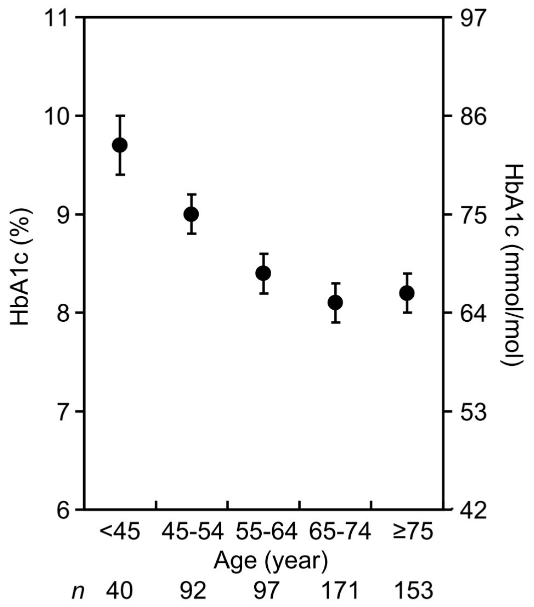

Supplementary Fig. S3: Receiver operating characteristics curve for PhA detecting diabetic microangiopathy.

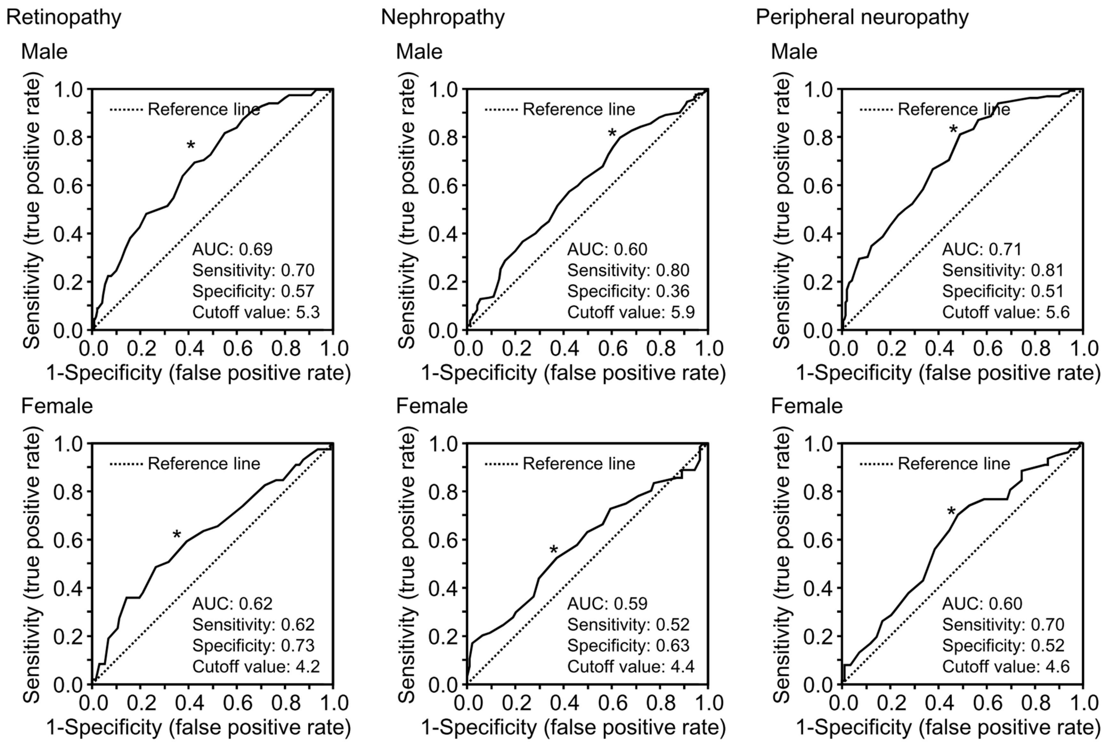

Supplementary Fig. S4: Relationship between PhA and SMI or ECW in SGLT2 inhibitor users and non-users.

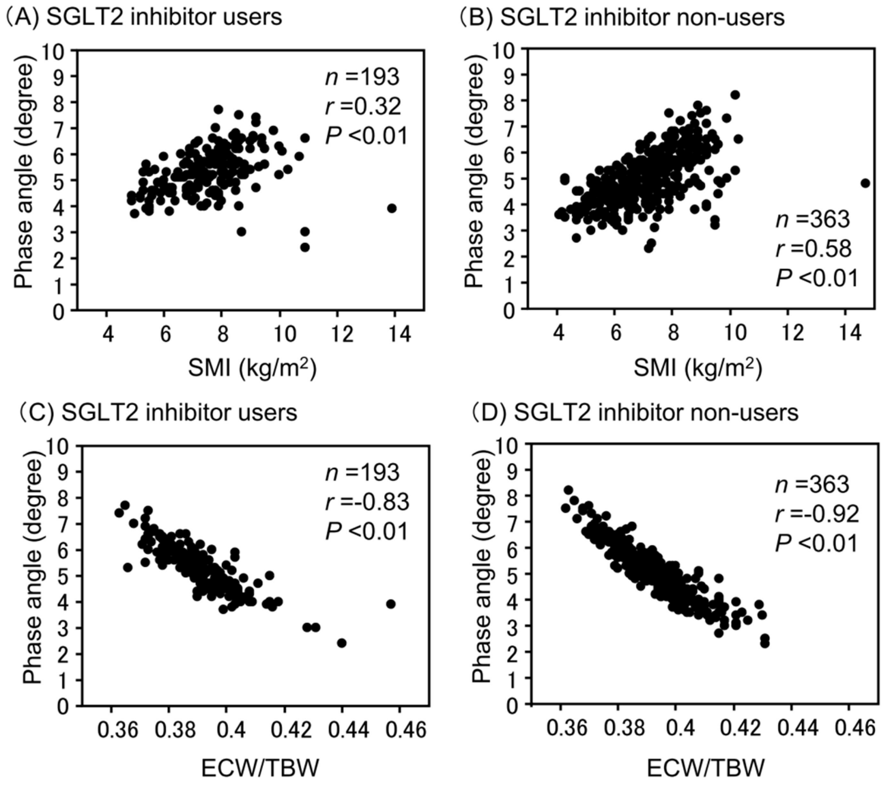

Supplementary Fig. S5: Relationships between the changes in SMI (A), ECW/TBW (B), and PhA (C), and the changes in HbA1c in 23 patients who underwent a second body composition evaluation.

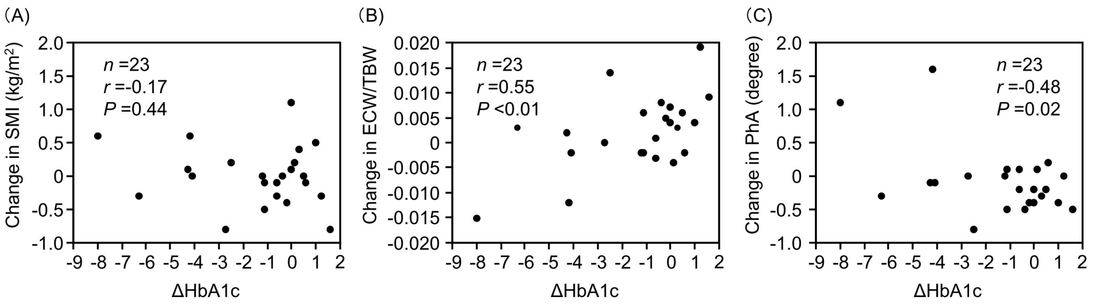

SMI, skeletal muscle index; ECW/TBW, extracellular water-to-total body water ratio; PhA, phase angle.
